# The Association of Parent-Child Communication With Internet Addiction in Left-Behind Children in China: A Cross-Sectional Study

**DOI:** 10.3389/ijph.2021.630700

**Published:** 2021-09-10

**Authors:** Jingjing Cai, Yun Wang, Feng Wang, Jingjing Lu, Lu Li, Xudong Zhou

**Affiliations:** ^1^ The Institute of Social and Family Medicine, School of Medicine, Zhejiang University, Hangzhou, China; ^2^ Department of Biomedical and Pharmaceutical Sciences, School of Pharmacy, Chapman University, California, CA, United States

**Keywords:** left-behind children, internet addiction, parent-child communication, paternal, maternal

## Abstract

**Objective: **Internet addiction has emerged as a growing concern worldwide. This study aimed to compare the prevalence of Internet addiction between left-behind children (LBC) and non-left-behind children (non-LBC), and explore the role of paternal and maternal parent-child communication on LBC.

**Methods: **We conducted a cross-sectional survey in rural areas in Anhui, China. The complete data were available from 699 LBC and 740 non-LBC. Multivariable logistic regression was used to examine 1) whether LBC were more likely to develop Internet addiction, and 2) the association between parent-child communication and Internet addiction among LBC.

**Results: **LBC had a higher likelihood to report Internet addiction when compared to non-LBC (OR = 2.03, 95%CI = 1.43–2.88, *p* < 0.001). Among LBC, parent-child communication (both mother-child and father-child) was protective factor for children’s Internet addiction. The role of mother-child communication played well among male LBC.

**Conclusions: **The lack of parental supervision may lead to Internet addiction. It is highly recommended for migrant parents to improve the quality of communication with their children. Also, gender-matching effects should be considered in the relationship between children’s behavior and parental factors.

## Introduction

The past few decades have witnessed rapid urbanization in China, with a sizable number of rural job hunters migrating from economically deprived areas to developed cities. Due to Chinese household registration systems [1], some social welfare services (e.g., free education at local public schools and coverage of medical insurance at local hospitals) are only provided to people with local “Hu Kou” (household registration at the municipal level) [2]. Thus, most migrant parents will leave their children behind in the place of original residence registration. Therefore their children are referred to in the literature as left-behind children (LBC) [3]. As estimated, in 2015, the number of LBC has nearly reached 69 million [4]. Previous studies found that parental absence would put children at risk for mental health disorders and problem behaviors [5,6]. A meta-analysis [7] demonstrated that LBC were more likely to develop depression, anxiety, suicidal ideation, conduct disorders, and substance use. It has been proven that parental migration may result in children’s unhealthy habits, and Internet addiction was one of problem behaviors LBC reported [8].

The term “Internet addiction” is defined as an obsessive state and uncontrolled internet use [9]. According to the China Internet Network Information Center (CINIC) data, as of June 2019, there were 854 million Chinese netizens, and children under 19 accounted for 20.9% [10]. Accompanying the increased internet and computer use, Internet addiction has increasingly been recognized as a serious, worldwide public health concern in recent decades. Recent evidence suggested that Internet addiction could contribute to psychiatric symptoms and behavioral problems, such as substance use disorder, attention-deficit hyperactivity disorder, social anxiety disorder, and suicidal attempts [11–13]. Moreover, the majority of LBC live with old-age grandparents [14]. Compared to parents, most grandparents have lower health literacy [15] and may lack proper parenting knowledge and skills to supervise and monitor the grandchildren [8]. The migrant parents, presumably out of the guilt for their absence, tend to compensate their children financially, such as more pocket money [16]. A lack of supervision and affordable internet access (for example, the internet café [17]) may facilitate Internet addiction behavior in LBC.

To date, there has been some disagreement on the variation in Internet addiction behaviour between LBC and non-left-behind children (non-LBC). One school-based survey in three Chinese counties, enrolling students from grade 7 to grade 9, revealed LBC (*n* = 593) were more likely to develop Internet addiction than non-LBC (*n* = 353) using a self-assessment questionnaire for adolescent problem behavior [18], while another cross-sectional study conducted in rural China, enrolling students from 8 to 17 years and using Young’s eight-item Internet addiction Scale, reported that LBC had a lower prevalence of Internet addiction (3.2%, *n* = 1,143) when compared to their rural counterparts (3.7%, *n* = 1,287) [19]. According to an urban-based survey where Adolescent Pathological Internet Use Scale was utilized, there exhibited no group difference among Chongqing secondary school students (*n* = 796) in the risk of Internet addiction [20]. Diverse study sites, assessment tools, sample characteristics, and sample size may contribute the variance in these findings. Given these contradictory results, there was a need to investigate whether LBC were more prone to Internet addiction than non-LBC or not.

Recent studies [14,21,22] emphasized the role of parent-child communication in mitigating adverse effects on LBC. Parent-child communication [23] refers to a dynamic procedure where family members can exchange their information and emotion. Good-quality parent-child communication enhances family cohesion and helps family members overcome troubles [24]. Despite the well-established association between parent-child communication and mental health in LBC [21,22], such as life satisfaction, loneliness, happiness, and depressive symptoms, most studies in the field of parent-child communication have only focused on the quantity and frequency of the contacts between parents and children or topics they touched on rather than the quality of the communication [21]. It has been well noted that both the quantity and quality of parent-child communication matter in alleviating adverse effects caused by parental migration [25].

Moreover, few research has looked at the potentially modifying role of parent-child communication in LBC’s Internet addiction behavior. The gender difference was also found for children’s Internet addiction and the quality of parent-child communication. A meta-analysis [26] of 119 independent samples from 34 countries/regions revealed that boys were more likely to have a higher level of Internet addiction than girls. Girls tend to be more active in conversation with parents than boys, and mothers seem likely to communicate with children than fathers. It seems that the association between parent-child communication and Internet addiction may vary from different gender-matching groups. In consideration of the gender-specific effect, there was a need to examine paternal and maternal communication with sons and daughters, respectively.

The current study aimed to compare the prevalence of Internet addiction between LBC and non-LBC and investigated the association between parent-child communication and Internet addiction among LBC. We hypothesize that gender-specific association would be found between parent-child communication and Internet addiction among the LBC community.

## Methods

### Participants and Procedure

We conducted a cross-sectional study in a school-based setting in Anhui Province in 2018. Anhui, a pioneering province in Southeastern China in the process of migration, had 16 million migrants for export in 2017 [27]. Participants were recruited using a stratified multistage sampling design. Considering the economic level and the density of migrant worker, two counties (Wuwei and Nanling) were chosen as study sites in the first stage. Two towns were selected randomly from each county and in each town, one elementary school and one middle school were selected randomly according to the school roster of the Town Education Bureau. In these eight participating schools, all students from grade 5 to grade 8 were included in this study. Children having reading and/or comprehension difficulties or reporting divorced, or deceased parent were excluded. Considering the complexity of the family process, especially the interaction between mother and father, children who live with both non-migrant parents and those who live with neither migrant parent (LBC) were defined as eligible participants.

Local permission to conduct this survey was obtained from local government, school principals or administrators. All eligible students and their guardians were provided with a detailed description of the study design and an informed consent form. Anonymous questionnaires were distributed to students who provided informed consent. Voluntary participation, withdrawn at any time, and the absence of a teacher ensured the anonymity of this investigation.

### Measures

#### Internet Addiction

Students’ Internet addiction behavior was assessed by using Young’s Internet Addiction Test for Chinese (YIAT-C) [28,29], a modified 20-item scale of Young’s Diagnostic Questionnaire. Students were asked to select one response option to a five-point Likert scale from “not at all” to “always.” The scores for all 20 items were summed (therefore from 20 to 100), and a total score of 50 or beyond meant a presence of the Internet addiction behavior [30]. The reliability and validity of YIAT-C have been established in Chinese children [6], and in the present study, the Cronbach’s alpha was 0.921.

#### Parental Migration Status

Paternal and maternal migration status and presence was examined by two questions respectively, “Has your father/your mother migrated into other places for work and been absent for over 6 months?”. The child was identified as LBC if his/her response to both questions were “Yes, currently absent,” and those living with both parents were considered as non-LBC.

#### Parent-Child Communication

The quality of parent-child communication was measured by the Chinese version Parent-Adolescent Communication Scale (PACS), which has been validated in the Chinese context [24]. PACS has 20 items, including two subscales of Openness of Parent–Adolescent Communication (10 items) and Problems in Parent–Adolescent Communication (10 items). Items were rated on a four-point Likert scale from 1 “strongly disagree” to 4 “strongly agree” to generate a total score and two subscale scores. A higher score indicates better parent-child communication. In the current study, the Cronbach’s alpha of paternal and maternal communication scales was 0.916 and 0.892, respectively.

#### Demographic Characteristics

The social-demographic characteristics included gender, age, grade, siblings, family income level, pocket money, and possession of a computer. Family income level was measured by subjective feelings of children because of the difficulty for children to judge the objective economic conditions of their families. The response item varies from “much poorer” to “much better” corresponding to their perception. Children were required to comment how much (1= “0 RMB/day,” 2= “1-6 RMB/day,” 3= “>6 RMB/day”) pocket money do they have a day.

### Statistical Analysis

We presented participants’ socio-demographic characteristics in counts and percentages. Mann-Whitney test (for continuous variables) and chi-square test (for categorical variables) were employed to examine demographic characteristics and Internet addiction among LBC and non-LBC. The multivariate logistic regressions after adjustment for covariates were performed to examine whether LBC carried a higher risk of developing Internet addiction. The covariates included grade, gender, possession of a computer, pocket money, any siblings and number of friends. Besides, interaction analyses of each covariate and parental migration status (LBC vs non-LBC) were tested to detect if the risk factors (e.g., gender) for Internet addiction differ statistically (with *p* < 0.05) between LBC and non-LBC. Finally, we conducted logistic regressions to explore the association between parent-child communication and Internet addiction among LBC, and to determine whether the effects of parent-child communication had a gender-specific pattern through the interaction term.

## Results

### Sample Characteristics

A total of 1,439 students agreed to participate in this study, of which 699 were LBC. Table 1 (insert Table 1 here) presents the social-demographic characteristics and Internet addiction. More than half of students were grade7 and grade8 (middle school) and boys. Approximately, two-thirds of respondents reported their family income level as “average.” The samples of LBC and non-LBC were comparable in terms of grade (*p* = 0.188), gender (*p* = 0.622), and family income level (*p* = 0.622), but LBC group reported significantly less ownership of computers than non-LBC group (*p* < 0.05). A higher proportion of (20.0%) LBC reported Internet addiction than non-LBC (12.2%, *p* < 0.001).

**TABLE 1:** Social demographic characteristics of rural children by parent migration status, n (%), a cross-sectional study conducted in Anhui Province, China, in 2018.

	LBC *N* = 699	Non-LBC *N* = 740	Z* or χ^2^	*p* value
Total				
Age, mean (SD)	12.4 (1.2)	12.5 (1.2)	0.596	0.551
Grade			1.73	0.188
Grade5 and Grade6	323 (46.2)	316 (42.8)		
Grade7 and Grade8	376 (53.8)	423 (57.2)		
Gender			0.244	0.622
Male	380 (55.0)	393 (53.7)		
Female	311 (45.0)	339 (46.3)		
Having any siblings			2.292	0.13
Yes	450 (64.6)	505 (68.3)		
No	247 (35.4)	234 (31.7)		
Friends number, mean (SD)	5.0 (3.3)	5.1 (3.2)	0.557	0.577
Self-rated family income level			0.95	0.622
Much better/better	193 (27.8)	205 (28.0)		
Average	455 (65.7)	470 (64.2)		
Much poorer/poorer	45 (6.5)	57 (7.8)		
Possession of a computer			4.656	0.031
No	350 (50.1)	329 (44.5)		
Yes	348 (49.9)	411 (55.5)		
Pocket money (RMB/day)			4.451	0.108
0	136 (19.5)	175 (23.8)		
1–6	308 (44.2)	295 (40.2)		
>6	253 (36.3)	264 (36.0)		
Internet addiction, *n* (%)			12.263	<0.001
No (IA group≤49)	424 (80.0)	495 (87.8)		
Yes (IA group≥50)	106 (20.0)	69 (12.2)		

Z, Mann-Whitney Test; SD, standard deviation; IA, Internet addiction; LBC, left-behind children.

### Association Between Parent Migration and Internet Addiction

Table 2 (insert Table 2 here) shows that parental migration is highly associated with Internet addiction, as well as other risk factors. After adjustments for socio-demographic characteristics, LBC were more than twice as likely to develop Internet addiction than non-LBC (OR = 2.03, 95%CI = 1.43–2.88, *p* < 0.001). We did not observe any significant interaction terms between parental migration and other risk factors, implying that these risk factors for Internet addiction were equally important for LBC and non-LBC.

**TABLE 2:** Parent migration and its risk factors in relation to Internet addiction, a cross-sectional study conducted in Anhui Province, China, in 2018.

	OR (95%CI)
Parental migration status	
Non-LBC	1
LBC	2.03 (1.43,2.88)^***^
Grade	
Grade5 and Grade6	1
Grade7 and Grade8	2.53 (1.71,3.73)^***^
*p* for interaction term	0.470
Possession of a computer	
No	1
Yes	1.44 (1.01,2.05)^*^
*p* for interaction term	0.213
Pocket money (RMB/day)	
0	1
1–6	1.67 (0.97,2.87)
>6	2.14 (1.25,3.65)^**^
*p* for interaction term	0.437
Gender	
Female	1
Male	1.47 (1.03,2.10)^*^
*p* for interaction term	0.806
Do you have any siblings?	
No	1
Yes	1.42 (0.97,2.08)
*p* for interaction term	0.708
Friends number	0.97 (0.94,1.01)
*p* for interaction term	0.615

CI, Confidence interval; OR, Odds ratio; LBC, left-behind children.

*: *p* < 0.05.

**: *p* < 0.01.

***: *p* < 0.001.

### Association Between Parent-Child Communication and Internet Addiction Among LBC

Table 3 (insert Table 3 here) reveals that mother- (OR = 0.95, 95%CI = 0.92–0.98, *p* < 0.001) and father-child communication (OR = 0.96, 95%CI = 0.94–0.99, *p* < 0.01) were both protective against Internet addiction among LBC. Compared with female LBC, mother-child communication showed significantly more effect on preventing male LBC from Internet addiction (OR = 0.92, 95%CI = 0.86–0.98, *p* < 0.01). What is interesting in this data is that there exists no gender difference in father-child communication.

**TABLE 3:** The association between parent-child communication and Internet addiction among left-behind children, a cross-sectional study conducted in Anhui Province, China, in 2018.

	Model 1 outcome: internet addiction OR (95%CI)	Model 2 outcome: internet addiction OR (95%CI)
MCC^a^	0.95 (0.92,0.98)^***^	0.99 (0.95,1.03)
FCC^b^	0.96 (0.94,0.99)^**^	0.94 (0.90,0.98)^**^
Grade		
Grade5 and Grade6	1	1
Grade7 and Grade8	2.23 (1.30,3.82)^**^	2.34 (1.36,4.05)^**^
Possession of a computer		
No	1	1
Yes	2.14 (1.31,3.49)^**^	2.11 (1.28,3.46)^**^
Gender		
Female	1	1
Male	1.31 (0.81,2.13)	11.75 (0.49,281.79)
MCC*Gender		
MCC*Female		1
MCC*Male		0.92 (0.86,0.98)^**^
FCC*Gender		
FCC*Female		1
FCC*Male		1.04 (0.99,1.10)

CI, Confidence interval; OR, Odds ratio.

*: *p* < 0.05.

**: *p* < 0.01.

***: *p* < 0.001.

a : Mother-child communication.

b : Father-child communication.

## Discussion

The uncontrolled use of the internet has led to disorders in psychological adjustments and health behaviors. Given the importance of parental company and supervision on preventing Internet addiction, this study aimed to explore the prevalence of Internet addiction and its risk factors across LBC and non-LBC. Based on our results, we have two main findings: 1) LBC were more vulnerable to Internet addiction; 2) Parent-child communication was protective against Internet addiction among LBC, and the association varied remarkably across gender of the parent and the child. Compared with father-child communication, the protective role of mother-child communication was specific to the child’s gender, where mother-son communication was more effective than mother-daughter communication.

Parental absence has been well acknowledged as a risk factors for child development [5,13]. In the present study, LBC were more likely to report Internet addiction. There are two possible psychological components to this issue: On the one hand, the internet provides children with an escape from negative events [5,31]. Some authors have speculated that [32], there existed unfulfilled emotional needs among LBC, and the internet has sometimes acted as a substitute for parents when children seek close contact and attention from the environment. Besides, the internet provided LBC with an approach to release their negative feelings. According to Young’s theory [29], the internet could serve as a typical mode of compensation for negative events in LBC’s lives, which means LBC may turn to internet to comfort themselves when drown in negative emotions (e.g., loneliness). On the other hand, a lack of parental supervision created an environment where children had easy internet access. And easy access without proper supervision may lead to a high likelihood of Internet addiction. The social control theory suggested parental supervision has a protective effect against adolescent problem behaviors [33], and the inadequate parental supervision was proven in literature as a risk factor for Internet addiction [21].

Our study also served as the first-hand evidence for the protective role of parent-child communication against LBC’s Internet addiction. A possible explanation for this might be that parent-child communication could attenuate Internet addiction through harmonious parent-child relationship. Good parent-child communication represented a good parent-child relationship [34], while a good parent-child relationship could effectively avoid Internet addiction among adolescents [35]. The other possible explanation might be the characteristics of good parent-child communication. Barnes and Olson [24] found that good communication with parents meant a free atmosphere of expression. In the context of mutual understanding, children will learn their emotional and social needs will be taken care of, which consequently reduces teens’ motivation to go online. Therefore, our study might suggest that migrant parents should maintain sustainable communication with their children by ensuring both the quantity and quality of communication. Parents may learn to improve communication skills and extend the conversation topics to create a supportive communication atmosphere and enhance the quality of parent-child communication.

Interestingly, our study identified a gender-specific influence of mother-child communication on Internet addiction: mother-child communication appeared to have a stronger effect on boys than girls. Traditionally, mothers take on more responsibilities as caregivers [36] and tend to be more active in family communication. Daughters and sons respond differently to the dominant and proactive role that mothers play. Liu et al. [37] pointed out girls might be keener to build social relationships than boys. Levin et al. [38] found girls were prone to look for other positive relationships to deal with poor communication with parents. It could be speculated that there are fewer ways for boys to build social bonds. When it comes to emotional expression and problem-solving, communicating with mothers might be more important for boys. Without this approach, boys are presumably more likely to turn to the internet, whereas girls may have more choices. The role of mothers in communication process [39] may be more critical for boys. However, the gender-specific effect was unobserved in father-child communication. Prior research [36] demonstrated that good communication with the father was associated with fewer suicidal behaviors in the female group than in the male group. This gender-specific pattern was not a one-size-fits-all case for all kinds of problem behaviors. Luk et al. [34] pointed out mother-child communication could prevent sons from smoking, which was not seen in girls. Kelly et al. [40] reported less frequent alcohol use was found only in female adolescents when an emotional relationship with the mother was close. Thus, it can be assumed that the protective association between family factors and adolescent problem behaviors might differ substantially across the gender (parents and children) and the type of behaviors. Considering diverse personality traits and behavioral preferences of males and females, it is recommended for further research to take gender-specific influence into account.

This study supplements the evidence of Internet addiction among LBC, expands our knowledge of protective role of high-quality parent-child communication in Internet addiction, and indicates gender-match patterns existing in family interactions. Several limitations, however, need to be acknowledged. First, the cross-sectional design limited our ability to draw a causal interpretation from our findings, especially the gender-specific influence. Our study firstly introduced gender-match patterns, among LBC group, into the association between parent-child communication and Internet addiction, and a longitudinal design is recommended for future studies to explore these issues. Second, we lacked the information on major caregivers and children’s living condition. Although we considered this information in the design process, it was difficult to connect their parents in our pre-investigation. Thirdly, this survey relied on self-reports which could introduce bias (e. g., social desirability bias, recall biases). Finally, this study was only conducted in two counties in the southeast of China, which required more caution in extrapolating these findings to the whole country.

Our study highlighted the high likelihood of Internet addiction in LBC community and suggested migrant parents keep good communication with their children. In order to help the transition from evidence to practice, we proposed four main suggestions to strengthen child protection, as well as reinforce the role of family in caring for left-behind children. First, for targeted policy development and implementation, government should lead an interdepartmental collaboration to figure out the amount of LBC, collect family information, and keep this information updated. Second, collaborative effort should be put into the examination and trajectory of bio-psycho-social health of children, especially those left behind. The partnership between educational and medical systems is encouraged to promote early detection and intervention of children’s health problems. Third, governments should pay more attention and give financial incentives to provide migrating parents with training programs to improve their communication skills with children. Last, governments could make efforts for short-term reunion of migrating families, for example, reunion in winter and summer vacation, which may directly enhance the communication between parents and children.

## Data Availability

The data-sets analyzed during this study are available from the corresponding authors on reasonable request.
